# Editorial: Rising stars in neuroradiology: 2022

**DOI:** 10.3389/fradi.2023.1349600

**Published:** 2024-01-05

**Authors:** Thomas C. Booth

**Affiliations:** ^1^ Department of Neuroradiology, Ruskin Wing, King’s College Hospital NHS Foundation Trust, London, United Kingdom; ^2^ School of Biomedical Engineering & Imaging Sciences, King’s College London, Rayne Institute, St. Thomas’ Hospital, London, United Kingdom

**Keywords:** deep learning, flow diverter, stroke, *T*_2_ relaxometry, NODDI, 18F-synvest-1, dual-source CT, cerebral vein thrombosis

**Editorial on the Research Topic** Rising stars in neuroradiology: 2022

In this Rising Stars in Neuroradiology: 2022 Research Topic, we highlight the diversity of research performed across the entire breadth of Neuroradiology and have presented advances in theory, experiment, and methodology with applications to compelling problems. In particular we focused on high-quality work of internationally recognized researchers in the early stages of their independent careers.

The Topic includes biomarker development studies incorporating CT, MRI, and PET as well as studies which will have the potential to improve cerebral revascularization and deep brain stimulation neurosurgery. Both preclinical and clinical methodology is also covered in the Topic as are insights into neuroradiology deep learning methodology.

Data from the Third International Stroke Trial (IST-3, a large randomized-controlled trial of intravenous alteplase for ischaemic stroke) is explored by Mair et al. for biomarker development. The authors first identified a cohort of patients who developed medium-large ischaemic lesions within 48 h based on a National Institutes of Health Stroke Scale (NIHSS) threshold. NIHSS > 11 best identified those with medium-large ischaemic lesions. The authors then explored whether the baseline CT of this cohort could be used as a predictive biomarker for thrombolysis outcome. Specifically, CT with normal appearing ischaemic brain tissue was compared to CT with visible lesions. They found that patients with invisible ischaemic lesions—and therefore a clinical-radiological mismatch—allocated to alteplase achieved more favourable outcome than those allocated to control. Prospective testing is now required but these exploratory analyses can plausibly inform the design of future studies where, for example, the time of stroke onset is unknown or outside conventional treatment time limits.

The development of quantitative magnetic resonance imaging (qMRI) methodology was considered by Fischi-Gomez et al. who evaluated three different techniques for estimating the *T*_2_ spectra from multi-echo *T*_2_ relaxometry in both gray and white matter. The results from their multicentre study suggest that the principal source of variance was the inter-subject variability as opposed to inter-site, inter-session, and inter-run variability. The implication is that multi-site neuroimaging studies can now develop complex tissue microstructure biomarkers by probing compartment-specific *T*_2_ relaxation times.

We shift gear towards the development of pre-clinical diagnostic biomarkers of the microbiome-gut-brain axis as proposed by Yi et al. In their pilot study, neurite orientation dispersion and density (NODDI) imaging, and SV2A 18F-SynVesT-1 synaptic density PET imaging is compared in mice with a functional gut microbiome and mice who are germ-free. Higher neurite density and orientation dispersion and decreased synaptic density was seen in the intact mice when compared to age- and sex-matched germ-free mice. Future multiparametric biomarker analyses will likely include metagenomic data as variables, prior to translational studies.

A clinically-orientated case-based overview focusing on the classic imaging characteristics of cerebral venous thrombosis and cerebral venous infarcts is included in this Topic (Li et al.). Of particular interest to readers is the description of the added value of magnetic resonance perfusion imaging and arterial spin-labelling. Future larger studies must now be performed to validate the diagnostic biomarkers described here. Such studies are important because when compared to arterial ischemic strokes, cerebral venous thrombosis has the potential for a more reversible course and favourable prognosis.

In another clinically-motivated article, dual-source CT systems were examined in single- and dual-energy modes by Zhao et al. The incentive for the study is that preoperative stereotactic planning of deep brain stimulation using CT may be degraded by frame-induced metal artefact which limits the visualization of adjacent brain structures. Using a cohort of patients with idiopathic Parkinson's disease, dual-energy modes (120 keV) provided better objective and subjective image quality at lower radiation doses compared to single-energy mode. Further studies comparing different CT systems would help in understanding the generalizability of the results.

The changing landscape of cerebral revascularization surgery in the context of interventional neuroradiology developments was explored by Gallagher et al. The authors described 50 cerebral revascularization procedures carried out over more than a decade. They explored whether, in the context of giant and fusiform cerebral aneurysms, flow-diverting stents have impacted on the use of cerebral revascularization surgery—and subsequently demonstrated trajectories for increasing and decreasing use of the two techniques, respectively. In this new era, where there is a reduction in cerebral revascularization surgical cases, the authors propose that in most European countries formal centralization of cerebral revascularization surgery will lead to the maintenance of high standards and good outcomes.

Optimising methods to label datasets for deep learning was investigated by Benger et al. The study was motivated by a lack of large clinically representative labelled datasets available to train neuroradiology deep learning models. The context of the study was a triage tool to determine whether brain MRIs were normal or abnormal—so that they can be flagged for rapid clinical reporting. The first experiment investigated a non-radiologist labelling strategy which was not pursued as there was a significant reduction in model performance accuracy compared to expert neuroradiologists. The second experiment investigated a junior radiologist labelling strategy which again was not pursued as there was a significant reduction in model performance accuracy. The third experiment explored report labelling as an expedient surrogate for image labelling. The authors showed that a report labelling strategy had a high sensitivity (98.7%), specificity (96.6%) and accuracy (98.5%) regarding separating normal and abnormal studies by leveraging Natural Language Processing (NLP) algorithms. The accuracy of more specific report labelling for multiple disease categories was shown to be variable and dependent on the category. The fourth experiment showed that a combination of axial *T*_2_-weighted sequences and those based on diffusion weighted imaging (DWI) were sufficient for training a computer vision triage tool, thereby limiting the number of MRI sequences needed as input. In summary, numerous lessons have been shared with the research community to help with deep learning methodology in neuroradiology.

We expect that the reader will find a useful range of clinically-orientated and methods-based studies in this state-of-the-art Research Topic.

